# Supplementary material for: Dietary Micronutrients and Risk of Chronic Kidney Disease: A Cohort Study with 12 Year Follow-Up
Source: Nutrients. 2021 Apr 30;13(5):1517. doi: 10.3390/nu13051517 (PMC8145051; doi:10.3390/nu13051517)
Supplement: Supplementary file 1 [file nutrients-13-01517-s001.zip › nutrients-1161788-supplementary.pdf]

Supplement Table S1. Dietary recommendation and average dietary intake of mineral and vitamin intakes for general population (KNHENAS 2001) and Ansan-Ansung cohort study participants (baseline 2001-2002; followed by 2014), a cohort of the KoGES (Korea Genome and Epidemiologic Study)

Diet intake	RDA, AI for general population	Dietary recommendation: dietary reference intakes (DRI) for CKD patients and supplementation[1]	KNHENAS 2001 Age 40-70	Ansan-Ansung cohort members with past CKD history [2]	Ansan-Ansung cohort members without past CKD history[2]
			GM (IQR)	GM (IQR)	GM (IQR)
**Calcium (mg)**	RDA: 1,300 mg	DRI: 800 mg (women CKD), 1000 mg (men CKD)<br>- CKD 3-4 patients not taking active vitamin D analogs: total elemental calcium intake of 800-1,000 mg/day (including dietary calcium, calcium supplementation and calcium-based phosphate binders)<br>- CKD 5D patients: adjust calcium intake	**434.3 (289.5-652.0)**	**445.0 (273.6-574.5)**	**466.0 (369.6-539.1)**
**Phosphorus (mg)**	RDA: 700 mg	- DRI: 400-700 mg (CKD)<br>- CKD 1-5D and post-transplant: phosphorus restriction treatment<br>- CKD 3-5 and on MHD: adjust dietary phosphorus intake to maintain serum phosphate levels in the normal range	1087.6 (824.5-1430.8)	968.3 (707.8-1153.5)	1066.9 (846.3-1234.4)
**Sodium (mg)**	AI: 2000 mg	- CKD 3-5 (non-dialyzed), dialysis, and post-transplant (1C): Limit sodium intake (< 100 mmol/day or <2.3 g/day) to reduce blood pressure and improve volume control.<br>- CKD 3-5 (non-dialyzed) with proteinuria: reduce sodium intake	**4912.7 (3324.2-7099.0)**	**3029.3 (1929.4-3829.3)**	**3116.9 (2472.5-3606.3)**
**Potassium(mg)**	AI: 3400 mg (AI)	- DRI: CKD 3-5D and post- transplant: adjust dietary potassium intake to maintain serum potassium within the normal range;<br>- CKD 3-5D and post-transplant with hyperkalemia: lower dietary potassium intake (eg. 2000 mg)<br>- CKD 3-5 on maintaining hemodialysis and post-transplant with either hyperkalemia or hypokalemia: dietary or supplemental potassium intake	**2790.6 (2084.7-3681.1)**	**2467.8 (1673.1-3000.5)**	**2576.2 (2043.6-2980.6)**
**Iron (mg)**	RDA: 8 mg (women aged ≥ 51 and adult men); 18 mg (women aged 19-50)	DRI: 10 mg (CKD); Regular blood test for iron overload (CKD)	11.4 (8.0-16.6)	10.5 (6.9-12.5)	11.1 (8.6-12.9)
**Zinc (mg)**	RDA: 8 mg	- DRI: 10 mg (men CKD); 8 mg (women CKD); 15 mg (Dialysis)<br>- Not routinely supplementing due to little evidence	N/A	8.4 (5.9-9.7)	9.0 (7.2-10.5)
**Vitamin A (R.E)**	RDA: 900 R.E	- DRI: 700-900 mg (CKD)]<br>- Not routinely supplement vitamin A or E because of the potential for vitamin toxicity	**501.4 (292.1-803.5)**	**504.4 (254.1-622.7)**	**490.0 (388.7-567.0)**
**Retinol**	-	-	23.7 (4.5-70.8)	57.7 (17.9-79.7)	51.7 (41.0-59.9)
**Carotene**	-	-	2497.1 (1455.1-4158.9)	2541.7 (1284.3-3093.9)	2415.5 (1916.1-2794.8)

**Vitamin E (mg)**	**<u>RDA: 11 mg (women); 15 mg (men)</u>**	- DRI: Up to RDA (CKD)<br>- Not routinely supplement vitamin A or E because of the potential for vitamin toxicity	N/A	**<u>8.9 (5.6-10.8)</u>**	**<u>9.4 (7.4-10.8)</u>**
**Vitamin B1 (mg)**	RDA: 1.1 mg	- DRI: 1.5 mg (CKD)	1.0 (0.8-1.5)	1.2 (0.8-1.4)	1.3 (1.0-1.5)
**Vitamin B2 (mg)**	RDA: 1.1 mg (women); 1,3 mg (men)	- DRI: 1.8 mg on a low-protein diet (CKD); 1.1-1.3 mg (dialysis), especially with poor appetite	1.0 (0.6-1.4)	1.0 (0.6-1.2)	1.0 (0.8-1.2)
**Niacin (mg)**	RDA: 14 mg (women); 16 mg (men)	- DRI: Near to RDA (Dialysis), 14-20 mg/day for CKD patients (dialysis and non-dialysis)	15.2 (10.6-21.8)	14.6 (10.4-17.3)	16.2 (12.8-18.7)
**Folate (mcg)**	**<u>RDA: 400 μg</u>**	- DRI: More than RDA (CKD)<br>- Stage 3-5 for hyperhomocysteinemia: not routinely supplementing<br>- Stage 1-5 for folic acid deficiency or insufficiency: recommend folate (1.0 mg/day), Vit B12 and/or B-complex supplement for CKD (dialysis and non-dialysis); Check B12 blood levels because folate supplements can mask a vitamin B12 deficiency.	N/A	**<u>240.8 (155.9-289.9)</u>**	**<u>246.4 (195.5-285.1)</u>**
**Vitamin B6 (mg)**	RDA: 1.3 mg	- DRI: 2 mg (Dialysis), 5 mg (Non-dialysis)<br>- Supplement: 10 mg (Dialysis) or 50 mg/day with folic acid and vitamin B12 to reduce homocysteine levels<br>- Large doses (200 mg/day) over long period can cause nerve damage.	N/A	1.7 (1.2-2.1)	1.9 (1.5-2.2)
**Vitamin C (mg)**	**<u>RDA: 75 mg (women); 90 mg (men)</u>**	- DRI: 60-100 mg (Dialysis)<br>- Stage 1-5 and post-transplant: 90mg supplement for men and 75mg for women CKD patients (dialysis and non-dialysis). Check serum oxalate levels (excess intake may cause oxalate deposits in bone and soft tissues).	**<u>113.7 (65.0-188.2)</u>**	**<u>120.2 (67.1-160.4)</u>**	**<u>116.8 (92.6-135.1)</u>**

Abbreviations: RDA, Recommended Dietary Allowance (average daily level of intake sufficient to meet the nutrient requirements of nearly all (97%-98%) healthy people); AI, Adequate Intake (established when evidence is insufficient to develop an RDA and is set at a level assumed to ensure nutritional adequacy). GM, Geometric mean; IQR, Inter-quartile range; KNHENAS, Korea National Health Examination and Nutritional Survey;

The underlined part means the case where the average dietary intake is low or high in the Korean general population or cohort members, compared to the RDA.

1. KDOQI Clinical practice guideline for nutrition in CKD
2. The subjects can be considered as those who were aware that they are CKD at the baseline. All of these subjects had an eGFR of less than 60 at baseline. Those were excluded from final study population. It could be seen that the subjects who were aware of CKD at baseline had increased vitamin A intake, while several minerals related to kidney function deterioration were lower than those of non-recognized subjects, compared to those who were unaware that they are transient or temporary CKD at the baseline. Vitamin intake among those was almost the same, except for vitamin A.
3. The subjects can be considered as those who were unaware that they are transient or temporary CKD at the baseline. All of these subjects had an eGFR of more than 60 at baseline. Those were included from final study population.

Supplementary Table S2. Baseline characteristics of study subjects in 9,079 Ansan-Ansung KoGES cohort population with 12-year follow-up

	No CKD [1]	New CKD cases at follow-up from 2 to 12 years				
		CKD cases[1]		CKD Stage 3A [1]	CKD Stage 3B and over [1]	
	**(N = 7,687)**	**(N = 1,392)**		**(N=1,239)**	**(N =153)**	
	**N (%)**	**N (%)**	***P***	**N (%)**	**N (%)**	***P***
Males	3867 (49.7)	492 (38.1)	<0.01	431 (37.8)	61 (39.9)	<0.01
Non-marriage [2]	644 (8.3)	181 (14.0)	<0.01	156 (13.7)	25 (16.3)	<0.01
Education ≥ high school	1097 (14.1)	119 (9.2)	<0.01	109 (9.6)	10 (6.5)	<0.01
Monthly income ≥ 3,000$	1455 (18.7)	131 (10.1)	<0.01	120 (10.5)	11 (7.2)	<0.01
No regular exercise [2]	3761 (48.3)	660 (51.0)	0.13	579 (50.8)	81 (52.9)	0.36
Alcohol drinkers	4371 (56.1)	543 (42.0)	<0.01	481 (42.2)	62 (40.5)	<0.01
Cigarette active smokers	3322 (42.7)	429 (33.2)	<0.01	370 (32.5)	59 (38.6)	<0.01
Passive smoking [2]	2987 (38.4)	410 (31.7)	<0.01	376 (33.0)	34 (22.2)	<0.01
Past history of						
Hypertension [3]	929 (11.9)	341 (26.4)	<0.01	280 (24.6)	61 (39.9)	<0.01
Diabetes [3]	393 (5.1)	189 (14.6)	<0.01	143 (12.5)	46 (30.1)	<0.01
Dyslipidemia	180 (2.3)	32 (2.5)	0.67	31 (2.7)	1 (0.6)	0.50
Gout	398 (5.1)	114 (8.8)	<0.01	97 (8.5)	17 (11.1)	<0.01
Myocardial infarction	52 (0.7)	12 (0.9)	0.42	8 (0.7)	4 (2.6)	0.06
Asthma	164 (2.1)	30 (2.3)	0.63	27 (2.4)	3 (2.0)	0.91
Allergy disease	422 (5.4)	73 (5.7)	0.68	66 (5.8)	7 (4.6)	0.88
Thyroid disease	235 (3.0)	40 (3.1)	0.71	39 (3.4)	1 (0.7)	0.37
Medication history						
Steroid	19 (0.2)	2 (0.1)	0.28	2 (0.0)	2 (0.2)	0.59
Insulin	40 (0.5)	35 (2.7)	<0.01	22 (1.9)	13 (8.5)	<0.01
Antihypertensive drugs	679 (8.7)	277 (21.4)	<0.01	277 (19.9)	50 (32.7)	<0.01
Levothyroxine	125 (1.6)	26 (2.0)	0.18	25 (2.2)	1 (0.7)	0.24
Diuretics	7 (0.1)	2 (0.2)	0.26	2 (0.2)	0 (0)	0.53
	**Mean (SD)**	**Mean (SD)**	***P***	**Mean (SD)**	**Mean (SD)**	***P***
Age (year)	50.7 (8.4)	59.2 (7.7)	<0.01	58.8 (7.7)	62.5 (6.3)	<0.01
Height (cm)	160.5 (8.6)	157.8 (8.4)	<0.01	157.9 (8.4)	156.9 (8.3)	<0.01
Weight (kg)	63.2 (10.1)	62.6 (10.2)	0.05	62.7 (10.2)	61.5 (10.4)	0.06
BMI (kg/m^2)	24.5 (3.1)	25.1 (3.3)	<0.01	25.1 (3.4)	24.9 (3.3)	<0.01
Waist (cm)	82.0 (8.6)	85.3 (8.9)	<0.01	85.1 (8.9)	86.6 (8.7)	<0.01
Hip size (cm)	93.5 (5.9)	93.8 (6.4)	0.09	93.9 (6.3)	92.7 (6.7)	0.01
SBP (mmHg)	119.3 (18.2)	128.3 (21.2)	<0.01	127.3 (20.9)	135.3 (22.0)	<0.01
DBP (mmHg)	79.3 (12.1)	82.6 (12.1)	<0.01	82.4 (12.1)	84.4 (12.0)	<0.01
Dietary intakes						
Energy (Cal/day)	1976.4 (724.8)	1893.7 (682.4)	<0.01	1899.9 (662.6)	1847.9 (816.4)	<0.01
Protein (g/day)	67.7 (31.3)	62.2 (26.6)	<0.01	62.6 (26.3)	59.0 (28.6)	<0.01
Carbohydrate (g/day)	345.7 (119.5)	343.8 (124.1)	0.59	344.0 (120.1)	342.2 (150.9)	0.85
Fat (g/day)	33.7 (22.3)	27.8 (17.2)	<0.01	28.2 (17.2)	25.2 (17.4)	<0.01
**Blood levels**						
Hemoglobin (g/dL)	13.6 (1.6)	13.4 (1.4)	<0.01	13.5 (1.4)	13.2 (1.4)	<0.01
Hematocrit (%)	41.1 (4.6)	40.6 (4.2)	<0.01	40.7 (4.3)	39.8 (4.1)	<0.01
WBC (10^3/μL)	6.6 (1.7)	6.5 (1.8)	0.08	6.6 (1.8)	6.8 (1.7)	0.12
Platelet (10^3/μL)	265.4 (64.2)	266.3 (67.3)	0.65	266.1 (65.0)	267.9 (82.9)	0.86
Cholesterol (mg/dL)	197.3 (36.4)	204.2 (38.1)	<0.01	204.1 (37.3)	204.4 (43.5)	<0.01
HDL (mg/dL)	49.8 (11.9)	48.1 (11.6)	<0.01	48.1 (11.6)	47.9 (11.9)	<0.01
Triglycerides (mg/dL)	148.5 (106.8)	175.6 (123.1)	<0.01	175.4 (125.6)	176.7 (102.6)	<0.01
FBS (mg/dL)	91.8 (21.5)	95.8 (29.5)	<0.01	94.8 (25.4)	105.1 (52.4)	<0.01
HbA1C (%)	5.7 (0.8)	6.2 (1.3)	<0.01	6.1 (1.2)	6.8 (2.0)	<0.01
AST (IU/L)	26.3 (20.1)	25.6 (13.0)	0.11	25.6 (13.1)	25.5 (12.9)	0.50
ALT (IU/L)	24.9 (27.2)	23.5 (17.1)	0.02	23.6 (17.6)	22.6 (12.9)	0.19
γ-GTP (mg/dL)	39.0 (71.1)	35.4 (70.7)	0.09	34.9 (71.9)	39.0 (61.2)	0.19
Total protein (g/dL)	7.30 (0.4)	7.26 (0.4)	0.02	7.3 (0.4)	7.2 (0.4)	<0.01
Albumin (g/dL)	4.51 (0.3)	4.44 (0.3)	<0.01	4.5 (0.3)	4.4 (0.3)	<0.01
hsCRP (mg/L)	1.37 (3.2)	1.95 (5.3)	<0.01	1.4 (3.2)	1.6 (2.6)	<0.01
Renin (ng/mL/hr)	2.7 (2.7)	2.7 (4.0)	0.51	2.6 (3.4)	3.9 (7.1)	<0.01
Creatinine (mg/dL)	0.80 (1.2)	0.84 (0.2)	<0.01	0.84 (0.2)	0.85 (0.2)	<0.01
BUN (mg/dL)	13.7 (3.6)	14.5 (3.6)	<0.01	14.3 (3.6)	15.5 (4.0)	<0.01
Calcium (mg/dL)	9.59 (0.5)	9.64 (0.5)	<0.01	9.6 (0.5)	9.6 (0.5)	<0.01
Sodium (mmol/L)	142.5 (2.2)	142.7 (2.1)	0.05	142.7 (2.1)	142.2 (2.5)	<0.01
Potassium (mmol/L)	4.48 (0.4)	4.51 (0.4)	<0.01	4.52 (0.41)	4.47 (0.4)	<0.01
Chloride (mmol/L)	103.0 (2.4)	103.0 (2.5)	0.60	103.1 (2.4)	102.7 (2.8)	0.11
Vitamin B12 (pg/mL)	681.6 (267.6)	697.2 (344.5)	0.69	687.2 (341.0)	757.2 (371.8)	0.64
Folate (ng/mL)	9.7 (7.1)	8.4 (6.0)	0.09	7.9 (5.0)	11.2 (10.1)	0.06
Free T4 (ng/mL)	1.2 (0.2)	1.2 (0.2)	0.98	1.2 (0.2)	1.1 (0.2)	0.70

TSH (μIU/ mL)	1.8 (2.0)	1.8 (1.5)	0.82	1.8 (1.6)	1.8 (0.9)	0.97
**Urine levels**						
Total protein (mg/dL)	8.3 (19.9)	9.2 (14.5)	0.26	8.5 (10.8)	14.5 (29.1)	0.01
Albumin (mg/dL)	2.1 (6.5)	3.0 (9.8)	0.06	2.5 (6.3)	7.2 (23.3)	<0.01
UPCR (g/g)	0.09 (0.22)	0.13 (0.24)	0.05	0.11 (0.18)	0.21 (0.46)	0.08
UACR (mg/g)	23.8 (77.9)	41.4 (74.9)	0.05	34.7 (124.3)	96.4 (390.8)	0.24
Microalbumin (μg/mL)	21.0 (10.6)	24.1 (54.1)	0.67	21.7 (48.6)	35.2 (74.4)	0.81
Creatinine (mg/dL)	109.2 (60.8)	95.2 (55.0)	<0.01	95.3 (54.2)	94.3 (60.7)	<0.01
Uric acid (mg/dL)	45.2 (20.0)	41.1 (19.4)	<0.01	41.1 (19.1)	40.7 (21.0)	<0.01
Calcium (mg/dL)	17.0 (23.6)	11.5 (8.4)	0.54	11.6 (8.3)	11.2 (9.5)	0.82
Sodium (mmol/L)	143.5 (53.8)	137.8 (48.0)	<0.01	137.2 (48.5)	141.3 (44.3)	0.03
Potassium (mmol/L)	52.5 (30.1)	53.3 (29.1)	0.51	53.1 (28.9)	54.8 (30.5)	0.72
PH	5.6 (0.8)	5.6 (0.8)	<0.01	5.6 (0.8)	5.5 (0.8)	0.03

Abbreviation: CKD, Chronic kidney disease; KoGES, Korea Genome and Epidemiologic Study; eGFR, Estimated glomerular filtration rate; CKD-EPI, Chronic kidney disease Epidemiology Collaboration; BMI, Body mass index; SBP, Systolic blood pressure; DBP, Diastolic blood pressure; eGFR, Estimated glomerular filtration rate; CKD-EPI, Chronic kidney disease Epidemiology Collaboration; WBC, White blood cell; HDL, High-density lipoprotein; FBS, Fasting blood sugar; HbA1C, Hemoglobin a1c; AST, Aspartate aminotransferase; ALT, Alanine aminotransferase; γ-GTP, γ-glutamyl transpeptidase; hsCRP, high-sensitivity C-reactive protein; BUN, Blood urea nitrogen; T4, Thyroxine; TSH, Thyroid stimulating hormone; UPCR, Urine Protein-to-Creatinine Ratio; UACR, Urine Albumin-to-Creatinine Ratio;

1. CKD was defined as an eGFR < 60 mL/min/1.73 m^2 by the CKD-EPI criteria. Stage 3A and Stage 3B and over were defined as 45≤eGFR<60 and eGFR<45, respectively.

2. Non-marriage means single, bereavement, and divorced status; Regular exercise was defined as performing regular exercise enough to sweat once a week or more; Passive smoking (among subjects who had never smoked) was determined by asking. "How many times do you indirectly inhale smoke from other people at home or your workplace?"

3. Hypertension was defined as a person with anti-hypertensive medication or systolic blood pressure ≥140, diastolic blood pressure ≥90 mm/Hg, or the presence of history of hypertension; Diabetes was defined as fasting blood glucose ≥126 mg/mL, or the presence of a history of diabetes.

Supplementary Table S3. Single micronutrient intake and CKD[1] risk in the restricted cohort population (N=8,901) [2]

	CKD stage 3A (N=987)			CKD stage 3B and over (N=128)			CKD (N=1,115)		
	**Person-years**	**New cases N**	**HR (95% CI) [3]**	**Person-years**	**New cases N**	**HR (95% CI) [3]**	**Person-years**	**New cases N**	**HR (95% CI) [3]**
**Calcium (mg)**									
< 200	6,142	105	1.0 (0.79-1.37)	5,425	22	1.9 (0.92-3.77)	6,274	127	1.1 (0.84-1.41)
200–400	27,017	405	1.1 (0.94-1.41)	24,168	45	1.2 (0.65-2.07)	27,275	450	1.1 (0.94-1.38)
400-600	21,924	249	0.9 (0.77-1.12)	20,149	32	1.1 (0.62-1.79)	22,117	281	0.9 (0.79-1.12)
≥ 600	19,054	228	1.0	17,454	29	1.0	19,236	257	1.0
**Phosphorus (mg)**									
< 400	851	13	0.8 (0.47-1.44)	809	10	**5.4 (2.59-11.37)**	908	23	1.2 (0.79-1.86)
400–700	12,874	215	1.0 (0.85-1.23)	11,334	32	**1.7 (1.00-2.77)**	13,078	247	1.1 (0.89-1.25)
700–1200	40,292	527	1.0	36,630	55	1.0	40,615	582	1.0
≥ 1200	20,120	232	0.8 (0.70-1.03)	18,423	31	0.8 (0.47-1.43)	20,303	263	0.8 (0.71-1.02)
**Sodium (mg)**									
< 2000	16,509	245	1.0 (0.84-1.19)	14,846	31	1.1 (0.66-1.87)	16,700	276	1.0 (0.85-1.19)
2000-2999	21,852	284	1.0	19,787	28	1.0	22,005	312	1.0
3000-3999	17,491	211	0.9 (0.79-1.13)	15,960	26	1.1 (0.61-1.82)	17,653	237	1.0 (0.80-1.13)
4000-4999	9,748	120	1.0 (0.77-1.19)	8,912	20	1.5 (0.84-2.77)	9,869	140	1.0 (0.82-1.23)
≥ 5000	8,538	127	1.1 (0.88-1.37)	7,691	23	1.8 (0.96-3.22)	8,677	150	1.1 (0.92-1.40)
**Potassium (mg)**									
< 1400	8,794	132	0.9 (0.67-1.19)	7,885	25	1.2 (0.41-3.68)	8,958	157	0.9 (0.68-1.16)
1400–2400	29,481	402	1.0 (0.78-1.23)	26,612	35	0.7 (0.30-1.73)	29,682	437	0.9 (0.74-1.14)
2400–3400	22,278	277	1.0 (0.85-1.25)	20,371	39	1.3 (0.69-2.40)	22,507	316	1.0 (0.86-1.24)
≥ 3400	13,583	176	1.0	12,328	29	1.0	13,756	205	1.0
**Iron (mg)**									
< 7	14,847	234	1.0 (0.85-1.22)	13,230	37	**1.8 (1.08-3.04)**	15,078	271	1.1 (0.90-1.26)
7–10	21,051	289	1.0	18,978	26	1.0	21,204	315	1.0
10–15	25,987	318	1.0 (0.79-1.16)	23,755	39	1.6 (0.89-2.96)	26,217	357	1.0 (0.83-1.21)
≥ 15	12,252	146	0.9 (0.71-1.18)	11,234	26	**2.1 (1.00-4.61)**	12,404	172	1.0 (0.78-1.26)
**Zinc (mg)**									
< 5	6,450	124	1.0 (0.72-1.28)	5,602	22	2.0 (0.92-4.41)	6,586	146	1.0 (0.78-1.34)
5–8	30,013	403	0.9 (0.69-1.08)	27,209	50	1.2 (0.66-2.38)	30,307	453	0.9 (0.72-1.10)
8–11	23,153	293	1.0	21,037	34	1.0	23,361	327	1.0
≥ 11	14,521	167	0.9 (0.72-1.06)	13,348	22	0.9 (0.49-1.50)	14,649	189	0.9 (0.73-1.05)
**Vitamin A (R.E)**									
< 300	18,967	312	1.1 (0.91-1.39)	16,807	42	0.9 (0.50-1.54)	19,212	354	1.1 (0.89-1.32)
300–500	24,030	311	1.0 (0.87-1.27)	21,800	30	0.6 (0.35-1.02)	24,210	341	1.0 (0.83-1.19)
500–700	14,397	173	1.1 (0.87-1.31)	13,120	22	1.0 (0.60-1.80)	14,520	195	1.1 (0.87-1.29)
≥ 700	16,743	191	1.0	15,469	34	1.0	16,961	225	1.0
**Retinol (μg)**									
< 20	13,488	271	1.2 (0.93-1.46)	11,693	50	**2.3 (1.20-4.25)**	13,776	321	1.3 (1.01-1.55)
20–60	25,439	337	1.2 (0.95-1.42)	22,926	36	1.5 (0.80-2.75)	25,638	373	1.2 (0.98-1.44)
60–100	18,470	210	1.1 (0.91-1.38)	17,029	26	1.4 (0.75-2.68)	18,644	236	1.2 (0.95-1.41)
≥ 100	16,740	169	1.0	15,548	16	1.0	16,845	185	1.0
**Carotene (μg)**									
< 1200	13,448	209	1.0 (0.78-1.20)	11,979	25	0.8 (0.42-1.39)	13,600	234	0.9 (0.77-1.15)
1200–2300	27,002	365	1.0 (0.86-1.24)	24,485	34	0.8 (0.46-1.23)	27,250	408	1.0 (0.85-1.19)
2300–3400	15,809	201	1.1 (0.87-1.28)	14,277	26	1.1 (0.64-1.84)	15,961	227	1.0 (0.87-1.26)
≥ 3400	17,879	212	1.0	16,456	34	1.0	18,092	246	1.0
**Vitamin E (mg)**									
< 5	10,973	175	0.9 (0.71-1.21)	9,799	29	1.0 (0.48-2.04)	11,137	204	0.9 (0.73-1.21)
5–8	22,636	335	1.1 (0.91-1.41)	20,206	31	0.8 (0.41-1.52)	22,828	366	1.1 (0.89-1.34)
8–11	19,154	232	1.0 (0.86-1.25)	17,534	35	1.2 (0.69-1.94)	19,352	267	1.1 (0.88-1.26)
≥ 11	21,374	245	1.0	19,656	33	1.0	21,586	278	1.0
**Vitamin B1 (mg)**									
< 0.9	17,897	288	1.0 (0.81-1.16)	15,901	39	1.1 (0.65-1.75)	18,141	327	1.0 (0.82-1.15)
0.9–1.2	18,447	259	1.0	16,680	30	1.0	18,622	289	1.0
1.2–1.5	19,071	221	1.0 (0.78-1.19)	17,506	27	1.3 (0.68-2.48)	19,231	248	1.0 (0.81-1.20)
≥ 1.5	18,721	219	0.9 (0.68-1.10)	17,109	32	1.3 (0.59-2.76)	18,910	251	0.9 (0.71-1.13)
**Vitamin B2 (mg)**									
< 0.7	17,716	319	1.1 (0.88-1.42)	15,565	49	**2.3 (1.13-4.82)**	18,019	368	1.2 (0.94-1.48)
0.7–0.9	18,632	245	1.0 (0.83-1.27)	16,886	30	1.7 (0.90-3.28)	18,791	275	1.1 (0.88-1.32)
0.9–1.2	19,130	228	1.0	17,462	20	1.0	19,253	248	1.0
≥ 1.2	18,659	195	0.9 (0.77-1.14)	17,284	29	1.2 (0.68-2.20)	18,840	224	1.0 (0.81-1.18)
**Niacin (mg)**									
< 10	17,926	293	0.7 (0.54-0.93)	10,488	33	1.0 (0.46-2.05)	18,211	339	0.7 (0.58-0.95)
10–14	18,491	256	0.8 (0.67-1.06)	19,300	36	0.8 (0.40-1.49)	18,661	286	0.9 (0.69-1.07)

14–19	18,813	240	1.0	20,898	36	1.0	18,972	267	1.0
≥ 19	18,906	189	0.9 (0.73-1.08)	16,510	23	0.9 (0.52-1.59)	19,059	223	0.9 (0.74-1.08)
**Folate (μg)**									
< 100	3,544	51	1.0 (0.72-1.53)	3,240	18	2.8 (0.90-8.56)	3,645	69	1.2 (0.84-1.64)
100–200	25,847	368	1.1 (0.88-1.50)	23,237	38	0.9 (0.37-2.41)	26,094	406	1.1 (0.84-1.37)
200–300	25,979	337	1.1 (0.86-1.39)	23,525	30	0.6 (0.28-1.25)	26,140	367	1.0 (0.82-1.27)
300–400	11,066	134	1.1 (0.87-1.47)	10,167	22	1.1 (0.59-2.12)	11,201	156	1.1 (0.89-1.44)
≥ 400	7,701	97	1.0	7,027	20	1.0	7,823	117	1.0
**Vitamin B6 (mg)**									
< 1.0	5,914	102	1.0 (0.78-1.27)	5,274	20	**2.4 (1.23-4.82)**	6,029	122	1.1 (0.86-1.34)
1.0–1.3	12,367	176	1.0 (0.81-1.21)	11,056	21	1.5 (0.78-2.98)	12,496	197	1.0 (0.83-1.23)
1.3–1.6	15,191	210	1.0	13,722	15	1.0	15,282	225	1.0
≥ 1.6	40,665	499	1.0 (0.83-1.25)	37,144	72	**2.3 (1.24-4.14)**	41,096	571	1.1 (0.90-1.34)
**Vitamin C (mg)**									
< 60	12,961	174	0.9 (0.76-1.17)	11,799	21	1.1 (0.58-2.27)	13,087	195	1.0 (0.78-1.18)
60–75	8,334	125	1.1 (0.89-1.41)	7,456	15	1.8 (0.85-3.68)	8,433	140	1.1 (0.92-1.43)
75–100	13,643	166	1.0	12,415	14	1.0	13,725	180	1.0
≥ 100	39,199	522	1.1 (0.89-1.28)	35,527	78	**1.9 (1.07-3.22)**	39,657	600	1.1 (0.94-1.32)

Abbreviation: CKD, Chronic kidney disease; KoGES, Korea Genome and Epidemiologic Study;
eGFR, Estimated glomerular filtration rate; CKD-EPI, Chronic kidney disease Epidemiology Collaboration

1. CKD was defined as an eGFR < 60 mL/min/1.73 m^2 by the CKD-EPI criteria. Stage 3A and Stage 3B and over were defined as 45≤eGFR<60 and eGFR<45, respectively.
2. Restricted cohort population excluding new CKD cases within 2 years from cohort entry
3. Cox proportional hazard model was constructed as Function(Y) = β_1[Single micronutrient] + 10 clinico-epidemiological confounders + 2 nutritional confounders + 1 additional confounders (if potassium); Function(Y) = Log $(\frac{\text{Hazard}_{\text{Exposed}}}{\text{Hazard}_{\text{Non-Exposed}}})$; 12 confounders were age, sex, baseline eGFR, body mass index (BMI), regular physical activity, cigarette smoking, alcohol consumption, total cholesterol level in blood, hypertension, diabetes, urine albumin to creatinine ratio, uric acid in urine, energy intake (mg/day), and protein intake (mg/day); For the analysis of ‘potassium intake’, additionally adjusted for folate intake/day.

Supplementary Table S4. Micronutrients and risk of CKD development (eGFR < 60) in the full multivariable model (Clinico-nutritional model)[2] controlling multiple nutrients and additional risk factors

	Entire cohort		Restricted cohort [3]	
	**HR (95% CI) [2]**	**P-value**	**HR (95% CI) [2]**	**P-value**
Age	1.10 (1.09-1.11)	<0.01	1.11 (1.10-1.11)	<0.01
Sex (Female)	1.77 (1.47-2.12)	<0.01	1.79 (1.47-2.18)	<0.01
Baseline eGFR	0.96 (0.95-0.96)	<0.01	0.96 (0.95-0.96)	<0.01
Smoker	1.17 (0.97-1.41)	0.10	1.21 (0.99-1.48)	0.06
Physical activity	0.79 (0.51-1.25)	0.31	0.96 (0.85-1.08)	0.48
BMI (≥ 25)	1.23 (1.10-1.38)	<0.01	1.22 (1.08-1.38)	<0.01
Hypertension	1.30 (1.16-1.45)	<0.01	1.30 (1.15-1.47)	<0.01
Diabetes	2.03 (1.75-2.35)	<0.01	2.14 (1.83-2.52)	<0.01
Diet energy intake	1.06 (0.85-1.31)	0.65	1.10 (0.91-1.33)	0.35
Diet protein intake	1.05 (0.85-1.31)	0.65	1.12 (0.89-1.42)	0.34
**Calcium (mg)** [4]				
< 200	1.08 (0.75-1.56)	0.67	1.07 (0.72-1.61)	0.73
200–400	1.12 (0.86-1.47)	0.39	1.20 (0.89-1.60)	0.23
400-600	0.92 (0.75-1.14)	0.45	0.94 (0.75-1.17)	0.57
≥ 600	1.0	-	1.0	-
**Phosphorus (mg)** [4]				
< 400	1.25 (0.76-2.06)	0.38	1.07 (0.61-1.86)	0.83
400–700	1.02 (0.79-1.31)	0.89	1.01 (0.77-1.33)	0.96
700–1200	1.0	-	1.0	-
≥ 1200	0.88 (0.69-1.11)	0.27	0.86 (0.67-1.11)	0.25
**Sodium (mg)** [4]				
< 2000	1.07 (0.90-1.27)	0.45	1.03 (0.85-1.24)	0.80
2000-2999	1.0	-	1.0	-
3000-3999	0.93 (0.79-1.10)	0.39	0.94 (0.79-1.13)	0.52
4000-4999	0.98 (0.79-1.21)	0.83	1.01 (0.81-1.27)	0.92
≥ 5000	1.21 (0.95-1.54)	0.12	1.25 (0.97-1.62)	0.08
**Iron (mg)** [4]				
< 7	1.00 (0.79-1.28)	0.98	1.05 (0.81-1.36)	0.72
7–10	1.0	-	1.0	-
10–15	0.96 (0.78-1.17)	0.67	0.93 (0.75-1.17)	0.54
≥ 15	1.04 (0.75-1.46)	0.80	1.02 (0.71-1.47)	0.92
**Retinol (µg)** [4]				
< 20	1.19 (0.92-1.54)	0.19	1.22 (0.93-1.61)	0.16
20–60	1.24 (0.99-1.54)	0.05	1.20 (0.95-1.52)	0.12
60–100	1.19 (0.98-1.45)	0.08	1.23 (1.00-1.52)	0.05
≥ 100	1.0	-	1.0	-
**Vitamin B2 (mg)** [4]				
< 0.7	1.04 (0.76-1.42)	0.79	1.22 (0.87-1.72)	0.24
0.7–0.9	0.99 (0.79-1.24)	0.96	1.04 (0.82-1.31)	0.77
0.9–1.2	1.0	-	1.0	-
≥ 1.2	0.99 (0.78-1.26)	0.94	0.96 (0.74-1.24)	0.75
**Folate (µg)** [4]				
< 100	1.10 (0.67-1.81)	0.71	1.34 (0.79-2.30)	0.28
100–200	1.09 (0.75-1.59)	0.66	1.09 (0.73-1.63)	0.28
200–300	1.10 (0.80-1.51)	0.56	1.00 (0.72-1.40)	0.99
300–400	1.27 (0.97-1.67)	0.09	1.15 (0.86-1.53)	0.34
≥ 400	1.0	-	1.0	-
**Vitamin B6 (mg)** [4]				
< 1.0	1.13 (0.80-1.60)	0.49	0.99 (0.68-1.45)	0.96
1.0–1.3	1.03 (0.82-1.30)	0.77	0.95 (0.75-1.22)	0.71
1.3–1.6	1.0	-	1.0	-
≥ 1.6	1.17 (0.93-1.47)	0.17	1.25 (0.98-1.59)	0.08
**Vitamin C (mg)** [4]				
< 60	0.85 (0.68-1.05)	0.14	0.82 (0.64-1.04)	0.10
60–75	1.00 (0.81-1.23)	0.99	1.06 (0.84-1.33)	0.64
75–100	1.0	-	1.0	-
≥ 100	1.06 (0.89-1.26)	0.48	1.21 (1.00-1.46)	0.05

Abbreviation: CKD, Chronic kidney disease; KoGES, Korea Genome and Epidemiologic Study; eGFR, Estimated glomerular filtration rate; CKD-EPI, Chronic kidney disease Epidemiology Collaboration.

1. CKD was defined as an eGFR < 60 mL/min/1.73 m² by the CKD-EPI criteria. Stage 3B and over were defined as eGFR<45, respectively.

2. Clinico-nutritional model was a multivariable Cox proportional hazard model, constructed as Function(Y) = $\sum_i^n$ βi[all variables] which were listed in the table; Function(Y) = Log $\left(\frac{\text{Hazard}_{\text{Exposed}}}{\text{Hazard}_{\text{Non-Exposed}}}\right)$

3. Restricted cohort analysis excluding new CKD cases within 2 years from cohort entry.

4. For calcium, RDA (recommended dietary allowance for general population) = 1300 mg; DRI (dietary reference intakes by KDOQI Clinical practice guideline for CKD patients or dialyzed CKD patients) = 800 mg (women CKD), 1000 mg (men CKD); For phosphorus, RDA = 700 mg; DRI = 400-700 mg (CKD); For sodium, AI (adequate intake for general population) = 2000 mg; DRI = limit or reduce sodium intake (CKD); For iron, RDA = 8 mg (women with age ≥ 51 or adult men), = 18 mg (women with age 19-50); DRI = 10 mg (CKD). CKD patients need regular blood tests for iron overload; For vitamin A precursor (retinol and carotene, not defined RDA or DRI); For vitamin B2, RDA = 1.1 mg (women) 1.3 mg (men), DRI: 1.8 mg on a low-protein diet (CKD), 1.1-1.3 mg (dialysis), especially with poor appetite; For folate, RDA = 400 µg, DRI = More than RDA (CKD); For vitamin B6, RDA = 1.3 mg, DRI = 2 mg (Dialysis); For vitamin C, RDA = 75 mg (women) 90 mg (men); DRI = 60-100 mg (dialysis).

Supplementary table S5. Micronutrients and CKD stages in the full multivariable model (Clinico-nutritional model)[2] controlling multiple nutrients and additional risk factors

CKD stage	Stage 3A		Stage 3B		Stage 4-5	
	Entire cohort		Entire cohort		Entire cohort	
	HR (95% CI) [3]	P-value	HR (95% CI) [3]	P-value	HR (95% CI) [3]	P-value
Age	1.10 (1.09-1.10)	<0.01	1.19 (1.15-1.23)	<0.01	1.16 (1.07-1.25)	<0.01
Sex (Female)	1.74 (1.43-2.11)	<0.01	2.56 (1.37-4.78)	<0.01	0.73 (0.11-4.74)	0.74
Baseline eGFR	0.96 (0.95-0.96)	<0.01	0.94 (0.93-0.95)	<0.01	0.94 (0.90-0.08)	<0.01
Smoker	1.09 (0.89-1.33)	0.39	1.91 (1.04-3.52)	0.04	3.16 (0.48-20.59)	0.23
Physical activity	0.98 (0.87-1.11)	0.78	1.33 (0.92-1.90)	0.13	0.71 (0.22-2.27)	0.56
BMI (≥ 25)	1.25 (1.10-1.41)	<0.01	1.43 (0.99-2.07)	0.06	0.44 (0.14-1.38)	0.16
Hypertension	1.28 (1.13-1.45)	<0.01	1.80 (1.26-2.55)	<0.01	1.34 (0.41-4.38)	0.63
Diabetes	1.82 (1.54-2.15)	<0.01	4.34 (2.94-6.41)	<0.01	35.02 (10.81-113.43)	<0.01
Diet energy intake			1.03 (0.57-1.86)	0.92	0.39 (0.09-1.65)	0.20
Diet protein intake	1.05 (0.84-1.31)	0.68	1.24 (0.60-2.54)	0.56	3.91 (0.39-39.18)	0.25
**Calcium (mg)** [1]						
< 200	1.21 (0.80-1.82)	0.37	0.57 (0.16-2.07)	0.39	18.68 (0.30-1159.54)	0.16
200–400	1.33 (0.98-1.80)	0.06	0.84 (0.32-2.22)	0.73	1.28 (0.08-19.82)	0.86
400-600	1.01 (0.80-1.27)	0.93	1.01 (0.49-2.09)	0.98	3.96 (0.60-26.29)	0.15
≥ 600	1.0	-	1.0	-	1.0	-
**Phosphorus (mg)** [4]						
< 400	0.86 (0.46-1.59)	0.64	8.72 (2.61-29.16)	<0.01	2.51 (0.06-115.331)	0.64
400–700	0.98 (0.75-1.28)	0.89	2.22 (0.96-5.15)	0.06	0.38 (0.02-6.78)	0.51
700–1200	1.0	-	1.0	-	1.0	-
≥ 1200	0.90 (0.70-1.16)	0.41	0.78 (0.34-1.79)	0.56	0.05 (0.00-0.67)	0.03
**Sodium (mg)** [1]						
< 2000	1.11 (0.93-1.33)	0.25	0.87 (0.47-1.61)	0.65	1.57 (0.26-9.55)	0.62
2000-2999	1.0	-	1.0	-	1.0	-
3000-3999	0.91 (0.77-1.09)	0.31	1.34 (0.77-2.34)	0.29	0.19 (0.03-1.13)	0.07
4000-4999	0.97 (0.77-1.21)	0.78	1.38 (0.72-2.65)	0.33	0.46 (0.06-3.78)	0.47
≥ 5000	1.23 (0.95-1.59)	0.11	1.50 (0.72-3.14)	0.28	1.03 (0.12-8.56)	0.97
**Iron (mg)** [1]						
< 7	0.96 (0.76-1.21)	0.72	0.96 (0.43-2.15)	0.91	2.16 (0.14-33.91)	0.58
7–10	1.0	-	1.0	-	1.0	-
10–15	0.94 (0.75-1.17)	0.56	1.16 (0.58-2.33)	0.68	1.29 (0.15-11.39)	0.82
≥ 15	0.95 (0.67-1.34)	0.76	1.63 (0.52-5.07)	0.40	26.81 (1.29-558.41)	0.03
**Retinol (μg)** [1]						
< 20	1.13 (0.86-1.49)	0.38	1.92 (0.82-4.45)	0.40	0.91 (0.07-10.67)	0.94
20–60	1.24 (0.98-1.56)	0.07	1.51 (0.71-3.25)	0.29	0.19 (0.02-1.88)	0.15
60–100	1.18 (0.96-1.45)	0.12	1.30 (0.65-2.59)	0.46	1.94 (0.42-8.89)	0.39
≥ 100	1.0	-	1.0	-	1.0	-
**Vitamin B2 (mg)** [1]						
< 0.7	0.94 (0.69-1.30)	0.72	2.54 (0.84-7.64)	0.10	52.09 (1.17-2328.86)	0.04
0.7–0.9	0.94 (0.75-1.19)	0.60	1.74 (0.80-3.75)	0.16	4.32 (0.38-49.20)	0.24
0.9–1.2	1.0	-	1.0	-	1.0	-
≥ 1.2	0.97 (0.77-1.23)	0.81	0.91 (0.40-2.04)	0.81	1.74 (0.22-13.85)	0.60
**Folate (μg)** [1]						
< 100	0.95 (0.56-1.63)	0.86	3.29 (0.74-14.65)	0.12	1.20 (0.01-133.82)	0.94
100–200	1.06 (0.71-1.58)	0.78	1.76 (0.54-5.72)	0.35	0.80 (0.02-35.04)	0.91
200–300	1.12 (0.79-1.57)	0.52	0.77 (0.31-1.91)	0.57	0.93 (0.06-15.13)	0.96
300–400	1.21 (0.90-1.63)	0.20	1.19 (0.56-2.54)	0.66	6.94 (0.75-63.93)	0.09
≥ 400	1.0	-	1.0	-	1.0	-
**Vitamin B6 (mg)** [1]						
< 1.0	1.10 (0.77-1.57)	0.59	0.96 (0.32-2.88)	0.94	0.09 (0.00-2.99)	0.08
1.0–1.3	1.05 (0.83-1.32)	0.71	0.95 (0.44-2.05)	0.89	0.07 (0.00-1.23)	0.07
1.3–1.6	1.0	-	1.0	-	1.0	-
≥ 1.6	1.13 (0.89-1.43)	0.31	3.16 (1.38-7.21)	<0.01	1.32 (0.15-11.96)	0.81
**Vitamin C (mg)** [1]						
< 60	0.88 (0.70-1.10)	0.27	0.76 (0.34-1.69)	0.50	0.39 (0.03-6.05)	0.50
60–75	0.98 (0.79-1.22)	0.83	1.46 (0.69-3.06)	0.32	6.41 (0.46-88.54)	0.17
75–100	1.0	-	1.0	-	1.0	-
≥ 100	1.03 (0.86-1.23)	0.75	1.70 (0.90-3.20)	0.10	5.63 (0.53-59.69)	0.15

Abbreviation: CKD, Chronic kidney disease; KoGES, Korea Genome and Epidemiologic Study; eGFR, Estimated glomerular filtration rate; CKD-EPI, Chronic kidney disease Epidemiology Collaboration.

1. 73 cases of CKD stage 3A and 25 cases of CKD 3B and over caused by both hypertension and diabetes.
2. Restricted cohort analysis excluding new CKD cases within 2 years from cohort entry.
3. Clinico-nutritional model was a multivariable Cox proportional hazard model, constructed as Function(Y) = $\sum_i^n$ βi[all variables] which were listed in the table; Function(Y) = Log ($\frac{\text{Hazard}_{\text{Exposed}}}{\text{Hazard}_{\text{Non-Exposed}}}$); UACR, urine uric acid

Supplementary Table S6. Single micronutrient intake and risk of diabetic and hypertensive CKD stage 3B and over in the entire cohort population (N=9,079) and restricted cohort population (N=8,901)

	**Risk of diabetic CKD stage 3B and over** [1]		**Risk of hypertensive CKD stage 3B and over** [1]	
	**Entire cohort**	**Restricted cohort** [2]	**Entire cohort**	**Restricted cohort** [2]
	New cases	New cases	New cases	New cases
	52	45	77	65
	HR (95% CI) [3]	HR (95% CI) [3]	HR (95% CI) [3]	HR (95% CI) [3]
**Calcium (mg)**				
< 200	**3.0 (1.01-9.10)**	***3.2 (0.98-10.55)***	2.3 (0.92-5.83)	2.3 (0.83-6.32)
200–400	0.6 (0.23-1.48)	0.6 (0.21-1.61)	1.1 (0.49-2.50)	1.4 (0.58-3.32)
400-600	1.1 (0.51-2.19)	1.0 (0.47-2.21)	1.4 (0.65-2.84)	1.4 (0.65-3.21)
≥ 600	1.0	1.0	1.0	1.0
**Phosphorus (mg)**				
< 400	**9.9 (1.91-51.06)**	**10.3 (1.92-55.53)**	**7.9 (3.46-18.20)**	**6.3 (2.29-17.13)**
400–700	**2.5 (1.02-6.07)**	2.5 (0.92-6.57)	1.2 (0.63-2.41)	1.4 (0.69-2.91)
700–1200	1.0	1.0	1.0	1.0
≥ 1200	1.0 (0.46-2.27)	1.1 (0.92-6.57)	0.7 (0.32-1.50)	0.6 (0.28-1.43)
**Sodium (mg)**				
< 2000	1.9 (0.76-4.82)	1.5 (0.54-4.04)	1.4 (0.73-2.65)	1.6 (0.76-3.24)
2000-2999	1.0	1.0	1.0	1.0
3000-3999	1.3 (0.52-3.42)	1.2 (0.42-3.29)	1.0 (0.46-1.96)	1.0 (0.44-2.27)
4000-4999	2.2 (0.83-5.93)	2.1 (0.73-6.06)	1.4 (0.64-3.03)	1.8 (0.77-4.08)
≥ 5000	**2.7 (1.05-7.20)**	**3.0 (1.12-8.26)**	1.3 (0.55-3.07)	1.5 (0.58-3.76)
**Potassium (mg)**				
< 1400	2.4 (0.34-17.35)	0.7 (0.08-5.75)	1.7 (0.38-7.87)	0.9 (0.18-4.71)
1400–2400	0.4 (0.08-2.22)	0.4 (0.08-2.23)	0.8 (0.22-2.64)	0.5 (0.13-1.94)
2400–3400	1.0 (0.41-2.26)	1.0 (0.41-2.64)	1.5 (0.66-3.63)	1.2 (0.47-3.22)
≥ 3400	1.0	1.0	1.0	1.0
**Iron (mg)**				
< 7	1.7 (0.67-4.52)	1.6 (0.57-4.52)	1.4 (0.77-2.64)	1.3 (0.68-2.66)
7–10	1.0	1.0	1.0	1.0
10–15	1.1 (0.38-2.96)	1.0 (0.33-3.03)	1.0 (0.47-2.22)	1.0 (0.44-2.39)
≥ 15	**3.5 (1.02-11.64)**	3.1 (0.86-11.33)	1.2 (0.44-3.45)	1.1 (0.36-3.37)
**Zinc (mg)**				
< 5	3.1 (0.87-11.26)	2.8 (0.69-11.31)	1.7 (0.62-4.76)	1.3 (0.41-3.81)
5–8	0.7 (0.24-1.87)	0.5 (0.17-1.75)	1.2 (0.52-2.95)	1.2 (0.48-2.97)
8–11	1.0	1.0	1.0	1.0
≥ 11	1.0 (0.47-2.20)	1.0 (0.45-2.38)	0.9 (0.43-2.04)	0.8 (0.36-1.88)
**Vitamin A (R.E)**				
< 300	0.5 (0.17-1.20)	0.4 (0.14-1.17)	1.4 (0.66-3.01)	1.7 (0.74-3.90)
300–500	0.4 (0.18-0.87)	0.4 (0.16-0.87)	0.9 (0.42-1.83)	1.0 (0.43-2.16)
500–700	1.1 (0.49-2.65)	1.0	1.2 (0.54-2.54)	1.4 (0.62-3.26)
≥ 700	1.0	1.2 (0.47-2.89)	1.0	1.0
**Retinol (μg)**				
< 20	1.6 (0.69-3.81)	1.5 (0.59-3.71)	**2.4 (1.06-5.24)**	**2.9 (1.19-6.92)**
20–60	0.8 (0.32-1.81)	0.7 (0.29-1.89)	1.1 (0.46-2.42)	1.2 (0.46-2.93)
60–100	0.8 (0.35-1.85)	0.9 (0.35-2.07)	1.1 (0.45-2.66)	1.4 (0.51-3.58)
≥ 100	1.0	1.0	1.0	1.0
**Carotene (μg)**				
< 1200	0.6 (0.21-1.73)	0.5 (0.15-1.56)	0.9 (0.38-1.99)	0.9 (0.38-2.36)
1200–2300	0.5 (0.23-0.99)	0.5 (0.22-1.03)	1.2 (0.62-2.37)	1.3 (0.64-2.77)
2300–3400	0.7 (0.27-1.72)	0.7 (0.25-1.80)	1.2 (0.60-2.56)	1.5 (0.68-3.26)
≥ 3400	1.0	1.0	1.0	1.0
**Vitamin E (mg)**				
< 5	0.8 (0.25-2.61)	0.8 (0.22-2.85)	1.4 (0.50-3.75)	1.8 (0.59-5.57)
5–8	0.3 (0.11-0.91)	0.4 (0.12-1.15)	1.0 (0.38-2.55)	1.5 (0.53-4.20)
8–11	0.5 (0.23-1.12)	0.5 (0.23-1.16)	1.3 (0.64-2.82)	2.0 (0.88-4.46)
≥ 11	1.0	1.0	1.0	1.0
**Vitamin B1 (mg)**				
< 0.9	1.3 (0.49-3.19)	1.3 (0.45-3.89)	1.6 (0.82-2.98)	1.4 (0.69-2.82)
0.9–1.2	1.0	1.0	1.0	1.0
1.2–1.5	1.7 (0.55-5.29)	3.2 (0.90-11.24)	1.5 (0.64-3.70)	1.5 (0.58-3.98)
≥ 1.5	2.6 (0.68-9.87)	**5.2 (1.16-23.63)**	1.2 (0.40-3.50)	1.3 (0.41-4.36)
**Vitamin B2 (mg)**				
< 0.7	1.8 (0.59-5.63)	1.5 (0.43-5.46)	1.8 (0.70-4.85)	2.5 (0.87-7.46)
0.7–0.9	0.9 (0.29-2.59)	0.8 (0.24-2.82)	0.9 (0.33-2.20)	1.3 (0.49-3.57)
0.9–1.2	1.0	1.0	1.0	1.0
≥ 1.2	1.4 (0.65-3.22)	1.7 (0.69-3.96)	0.9 (0.42-1.97)	0.9 (0.40-2.17)
**Niacin (mg)**				
< 10	2.1 (0.51-8.68)	1.8 (0.39-8.75)	0.6 (0.23-1.69)	0.6 (0.20-1.63)

10–14	1.3 (0.38-4.22)	1.1 (0.30-4.19)	0.7 (0.27-1.59)	0.5 (0.21-1.36)
14–19	1.0	1.0	1.0	1.0
≥ 19	1.5 (0.69-3.44)	1.4 (0.59-3.24)	0.9 (0.41-1.90)	1.0 (0.43-2.20)
**Folate (μg)**				
< 100	0.7 (0.05-10.43)	1.0 (0.06-16.87)	2.6 (0.61-11.11)	2.9 (0.62-13.44)
100–200	0.4 (0.04-3.54)	0.4 (0.04-4.05)	0.8 (0.21-2.65)	0.9 (0.24-3.20)
200–300	0.6 (0.22-1.79)	0.5 (0.17-1.72)	0.7 (0.25-1.84)	0.8 (0.27-2.11)
300–400	1.0 (0.42-2.39)	0.9 (0.34-2.21)	1.6 (0.66-4.02)	1.1 (0.43-3.08)
≥ 400	1.0	1.0	1.0	1.0
**Vitamin B6 (mg)**				
< 1.0	**5.4 (1.55-19.11)**	**5.3 (1.27-22.44)**	**2.4 (1.10-5.24)**	2.2 (0.90-5.33)
1.0–1.3	1.5 (0.37-6.27)	2.2 (0.48-10.41)	0.9 (0.40-2.23)	1.2 (0.48-2.94)
1.3–1.6	1.0	1.0	1.0	1.0
≥ 1.6	5.3 (1.48-18.63)	6.7 (1.60-28.45)	1.7 (0.75-3.98)	1.8 (0.73-4.65)
**Vitamin C (mg)**				
< 60	1.0 (0.26-3.77)	0.6 (0.12-2.55)	1.4 (0.62-3.19)	1.1 (0.45-2.77)
60–75	2.3 (0.51-10.72)	2.3 (0.51-10.57)	1.9 (0.75-4.64)	2.2 (0.85-5.62)
75–100	1.0	1.0	1.0	1.0
≥ 100	3.2 (1.10-9.43)	2.4 (0.81-7.22)	1.4 (0.66-3.99)	1.3 (0.56-2.84)

Abbreviation: CKD, Chronic kidney disease; KoGES, Korea Genome and Epidemiologic Study; eGFR, Estimated glomerular filtration rate; CKD-EPI, Chronic kidney disease Epidemiology Collaboration.

1. 73 cases of CKD stage 3A and 25 cases of CKD 3B and over caused by both hypertension and diabetes.

2. Restricted cohort analysis excluding new CKD cases within 2 years from cohort entry.

3. Cox proportional hazard model was constructed as Function(Y) = β_1[Single micronutrient] + 10 clinico-epidemiological confounders + 2 nutritional confounders + 1 additional confounders (if potassium); Function(Y) = Log ($\frac{\text{Hazard}_{\text{Exposed}}}{\text{Hazard}_{\text{Non-Exposed}}}$); 12 confounders were age, sex, baseline eGFR, body mass index (BMI), regular physical activity, cigarette smoking, alcohol consumption, total cholesterol level in blood, hypertension, diabetes, urine albumin to creatinine ratio, uric acid in urine, energy intake (mg/day), and protein intake (mg/day); For the analysis of ‘potassium intake’, additionally adjusted for folate intake/day.

Supplementary Table S7. Micronutrients and risk of diabetic and hypertensive CKD stage 3B and over in the full multivariable model (Clinico-nutritional model) controlling multiple nutrients and additional risk factors

	**Risk of diabetic CKD stage 3B and over** [1]				**Risk of hypertensive CKD stage 3B and over** [1]			
	**Entire cohort**		**Restricted cohort** [2]		**Entire cohort**		**Restricted cohort** [2]	
	**HR (95% CI)** [3]	**P-value**	**HR (95% CI)** [3]	**P-value**	**HR (95% CI)** [3]	**P-value**	**HR (95% CI)** [3]	**P-value**
Age	1.15 (1.09-1.21)	<0.01	1.15 (1.09-1.21)	<0.01	1.17 (1.11-1.22)	<0.01	1.16 (1.11-1.22)	<0.01
Sex (Female)	1.70 (0.51-5.64)	0.38	1.94 (0.57-6.65)	0.29	2.05 (0.82-5.10)	0.12	1.75 (0.69-4.42)	0.23
Baseline eGFR	0.96 (0.93-0.98)	<0.01	0.98 (0.951.01)	0.13	0.92 (0.90-0.94)	<0.01	0.93 (0.91-0.95)	<0.01
Smoker	2.11 (0.70-6.34)	0.18	1.38 (0.44-4.38)	0.58	1.64 (0.68-3.99)	0.27	1.24 (0.50-3.10)	0.64
Physical activity	2.82 (1.35-5.89)	<0.01	2.26 (1.04-4.91)	0.03	0.87 (0.52-1.43)	0.57	0.83 (0.48-1.44)	0.51
BMI (≥ 25)	0.54 (0.27-1.06)	0.07	0.51 (0.24-1.05)	0.06	1.11 (0.66-1.87)	0.69	1.06 (0.60-1.87)	0.83
Hypertension	1.22 (0.63-2.38)	0.55	1.35 (0.66-2.80)	0.41	6.73 (3.87-11.71)	<0.01	7.88 (4.37-14.21)	<0.01
Diet energy intake	0.49 (0.18-1.32)	0.16	0.52 (0.18-1.51)	0.23	1.02 (0.45-2.29)	0.97	0.93 (0.39-2.25)	0.87
Diet protein intake	0.96 (0.25-3.51)	0.95	0.56 (0.14-2.30)	0.42	1.65 (0.59-4.63)	0.35	1.82 (0.59-5.69)	0.30
**Calcium (mg)** [1]								
< 200	2.44 (0.17-34.71)	0.51	10.07 (0.44-232.16)	0.14	0.65 (0.09-4.52)	0.65	0.47 (0.06-3.71)	0.47
200–400	0.94 (0.15-6.00)	0.95	2.24 (0.31-16.43)	0.42	0.79 (0.19-3.31)	0.74	0.71 (0.16-3.16)	0.64
400-600	2.11 (0.72-6.21)	0.18	3.22 (0.99-10.48)	0.05	1.30 (0.47-3.59)	0.61	1.26 (0.42-3.78)	0.67
≥ 600	1.0	-	1.0	-	1.0	-	1.0	-
**Phosphorus (mg)** [1]								
< 400	13.59 (0.60-308.39)	0.10	12.83 (0.48-343.93)	0.12	15.5 (3.28-73.61)	<0.01	13.3 (2.33-76.44)	<0.01
400–700	9.12 (0.85-97.58)	0.06	7.69 (0.63-93.49)	0.11	1.72 (0.56-5.24)	0.34	2.01 (0.62-6.51)	0.25
700–1200	1.0	-	1.0	-	1.0	-	1.0	-
≥ 1200	0.36 (0.10-1.28)	0.11	0.46 (0.13-1.65)	0.23	0.31 (0.17-1.76)	0.05	0.32 (0.09-1.14)	0.07
**Sodium (mg)** [1]								
< 2000	1.19 (0.34-3.24)	0.78	1.10 (0.58-2.32)	0.44	1.60 (0.66-3.84)	0.29	2.35 (0.95-5.86)	0.06
2000-2999	1.0	-	1.0	-	1.0	-	1.0	-
3000-3999	0.56 (0.18-1.76)	0.32	0.53 (0.15-1.90)	0.33	0.89 (0.40-1.99)	0.76	1.06 (0.43-2.61)	0.89
4000-4999	0.84 (0.24-3.00)	0.79	0.94 (0.23-3.81)	0.93	1.26 (0.50-3.17)	0.62	2.16 (0.82-5.74)	0.12
≥ 5000	2.00 (0.55-7.26)	0.29	2.02 (0.52-7.90)	0.31	1.03 (0.36-2.98)	0.95	1.56 (0.50-4.90)	0.44
**Iron (mg)** [1]								
< 7	0.23 (0.03-1.74)	0.16	0.20 (0.02-1.80)	0.15	0.55 (0.17-1.76)	0.31	0.55 (0.17-1.85)	0.33
7–10	1.0	-	1.0	-	1.0	-	1.0	-
10–15	0.41 (0.11-1.57)	0.19	0.53 (0.12-2.46)	0.41	0.72 (0.29-1.78)	0.47	0.84 (0.31-2.24)	0.72
≥ 15	2.21 (0.32-15.46)	0.42	2.18 (0.27-17.47)	0.46	1.36 (0.30-6.26)	0.69	1.42 (0.27-7.57)	0.68
**Retinol (μg)** [1]								
< 20	0.60 (0.14-2.83)	0.55	0.57 (0.11-3.02)	0.50	2.72 (0.86-8.63)	0.08	3.78 (1.09-13.06)	0.03
20–60	0.55 (0.17-1.76)	0.31	0.48 (0.13-1.76)	0.27	1.38 (0.49-3.88)	0.54	1.51 (0.48-4.72)	0.47
60–100	0.45 (0.17-1.21)	0.11	0.59 (0.20-1.70)	0.32	1.02 (0.38-2.73)	0.96	1.38 (0.47-4.01)	0.55
≥ 100	1.0	-	1.0	-	1.0	-	1.0	-
**Vitamin B2 (mg)** [1]								
< 0.7	3.96 (0.39-40.15)	0.24	1.56 (0.12-21.15)	0.73	2.61 (0.48-14.13)	0.96	2.78 (0.43-17.96)	0.28
0.7–0.9	1.65 (0.39-6.91)	0.49	1.18 (0.24-5.77)	0.83	0.83 (0.27-2.58)	0.26	1.06 (0.31-3.61)	0.93
0.9–1.2	1.0	-	1.0	-	1.0	-	1.0	-
≥ 1.2	0.87 (0.28-2.71)	0.81	1.39 (0.40-4.88)	0.60	1.04 (0.35-3.09)	0.93	1.13 (0.35-3.62)	0.84
**Folate (μg)** [1]								
< 100	0.20 (0.01-4.22)	0.30	0.76 (0.03-22.35)	0.87	1.79 (0.25-12.59)	0.55	4.03 (0.47-35.01)	0.20
100–200	0.45 (0.04-5.45)	0.53	0.48 (0.03-6.60)	0.58	0.91 (0.20-4.21)	0.90	1.03 (0.21-5.15)	0.97
200–300	0.60 (0.14-2.53)	0.49	0.38 (0.08-1.69)	0.20	0.71 (0.20-2.47)	0.59	0.77 (0.21-2.84)	0.71
300–400	1.76 (0.61-5.10)	0.30	1.24 (0.41-3.78)	0.69	1.54 (0.54-4.36)	0.42	1.05 (0.34-3.24)	0.93
≥ 400	1.0	-	1.0	-	1.0	-	1.0	-
**Vitamin B6 (mg)** [1]								
< 1.0	2.23 (0.21-23.47)	0.50	1.52 (0.11-20.45)	0.75	0.59 (0.12-2.89)	0.51	0.42 (0.07-2.58)	0.35
1.0–1.3	0.59 (0.08-4.36)	0.61	1.12 (0.15-8.54)	0.91	0.79 (0.28-2.22)	0.66	0.89 (0.30-2.62)	0.83
1.3–1.6	1.0	-	1.0	-	1.0	-	1.0	-
≥ 1.6	5.98 (1.16-30.78)	0.03	10.48 (1.36-80.84)	0.02	2.27 (0.76-6.82)	0.14	2.80 (0.87-9.07)	0.08
**Vitamin C (mg)** [1]								
< 60	0.41 (0.09-1.87)	0.25	0.23 (0.04-1.35)	0.10	0.70 (0.23-2.11)	0.53	0.56 (0.17-1.87)	0.34
60–75	0.46 (0.07-3.05)	0.42	0.42 (0.06-2.98)	0.38	1.42 (0.53-3.82)	0.48	1.71 (0.62-4.77)	0.29
75–100	1.0	-	1.0	-	1.0	-	1.0	-
≥ 100	2.51 (0.70-9.00)	0.16	1.53 (0.41-5.73)	0.53	1.50 (0.64-3.54)	0.35	1.35 (0.55-3.32)	0.51

Abbreviation: CKD, Chronic kidney disease; KoGES, Korea Genome and Epidemiologic Study; eGFR, Estimated glomerular filtration rate; CKD-EPI, Chronic kidney disease Epidemiology Collaboration.

4. 73 cases of CKD stage 3A and 25 cases of CKD 3B and over caused by both hypertension and diabetes.

5. Restricted cohort analysis excluding new CKD cases within 2 years from cohort entry.

6. Clinico-nutritional model was a multivariable Cox proportional hazard model, constructed as Function(Y) = $\sum_i^n$ βi[all variables] which were listed in the table; Function(Y) = Log ($\frac{\text{Hazard}_{\text{Exposed}}}{\text{Hazard}_{\text{Non-Exposed}}}$); UACR, urine uric acid

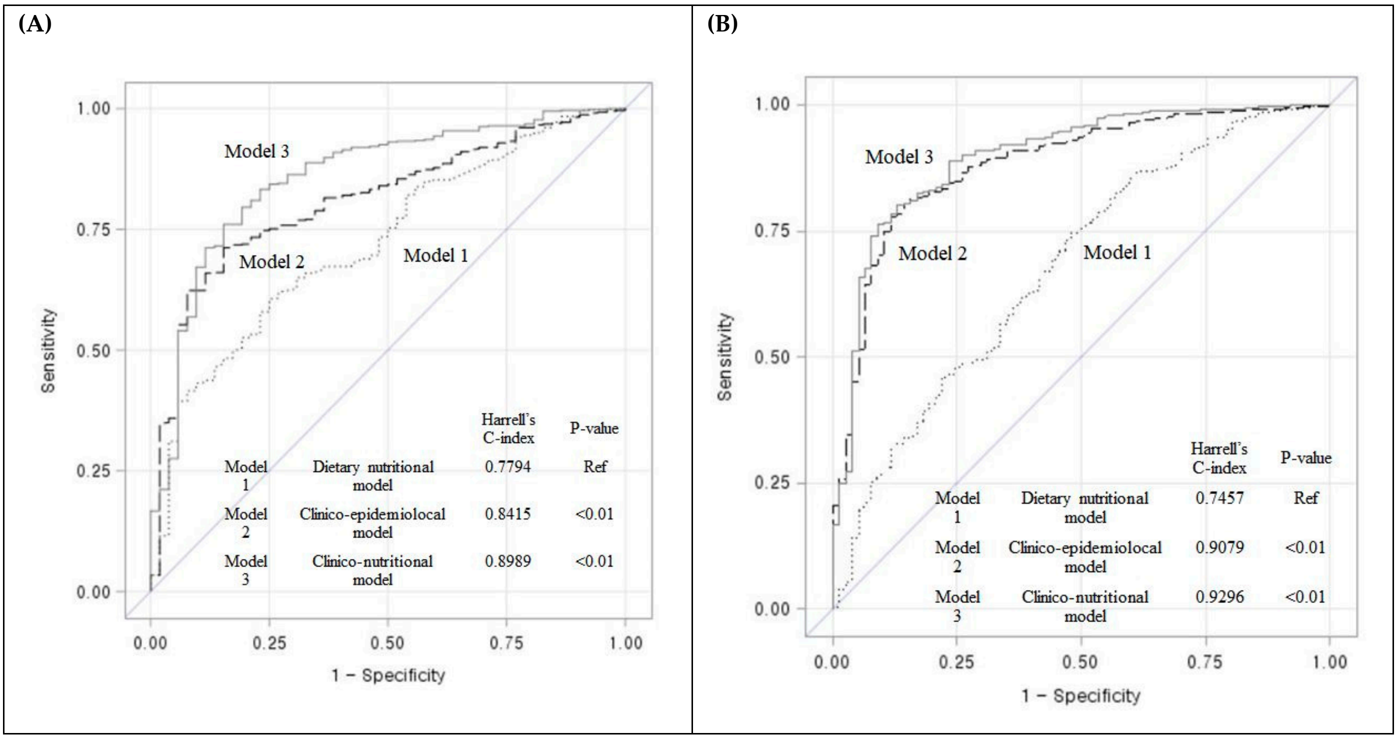

Supplementary Figure S1. Receiver operating characteristic (ROC) curves and Harrell's C-index showing the discriminant accuracy of each model for the ability to distinguish new cases of diabetic or hypertensive CKD stage 3B and over in entire cohort population over 12-year follow-up periods: [A] diabetic CKD stage 3B and over [B] hypertensive CKD stage 3B and over (Abbreviation: CKD, Chronic kidney disease. Model 1 (Dietary Nutritional Model): Function(Y) = β_1[Calcium] + β_2[Phosphorus] + β_3[Sodium] + β_4[Iron] + β_5[Retinol] + β_6[Vitamin B2] + β_7[Folate] + β_8[Vitamin B6] + β_9[Vitamin C] + β_{10}[Total Calories] + β_{11}[Protein], Function(Y) = Log $\left(\frac{\text{Hazard}_{\text{Exposed}}}{\text{Hazard}_{\text{Non-Exposed}}}\right)$; Model 2 (Clinico-epidemiolocal model): Function(Y) = β_1[Age] + β_2[Sex] + β_3[Baseline eGFR] + β_4[Physical activity] + β_5[Cigarette smoking] + β_6[Hypertension] + β_7[Diabetes] + β_8[Body mass index]; Model 3 (Clinico-nutritional model, a composite model of Model 1 and Model 2): Function(Y) = β_{11}^{1}[Model 1] + β_{8}^{1}[Model 2]).
